# Prognostic impact of *RUNX1* mutations and deletions in pediatric acute myeloid leukemia: results from the French ELAM02 study group

**DOI:** 10.1038/s41375-023-01931-y

**Published:** 2023-06-16

**Authors:** Lucille Lew-Derivry, Alice Marceau-Renaut, Laurène Fenwarth, Wendy Cuccuini, Paola Ballerini, Maxime Ferreboeuf, Audrey Guilmatre, Arnaud Petit, Virginie Gandemer, Fanny Rialland, Pascale Schneider, Gérard Michel, Yves Bertrand, Andre Baruchel, Claude Preudhomme, Guy Leverger, Hélène Lapillonne

**Affiliations:** 1 AP-HP, Pediatric Hematology and Oncology Department, Trousseau Hospital, F-75012 Paris, France; 2 CHU Lille, Laboratory of Hematology, F-59000 Lille, France; 3 INSERM, UMR-S 1172, F-59000 Lille, France; 4 AP-HP, Department of cytogenetics, Saint-Louis Hospital, F-75010 Paris, France; 5 AP-HP, Laboratory of Hematology, Trousseau hospital, F-75012 Paris, France; 6 Sorbonne Université, INSERM, UMRS_938, Centre de Recherche Saint-Antoine-CRSA, F-75012 Paris, France; 7 Department of Pediatric Hematology/Oncology, University Hospital of Rennes, F-35000 Rennes, France; 8 Department of Pediatric Hematology/Oncology, University Hospital of Nantes, F-44000 Nantes, France; 9 Department of Pediatric Hematology/Oncology, University Hospital Charles-Nicolle, F-76000 Rouen, France; 10 AP-HM, Department of Pediatric Hematology, La Timone University Hospital, F-13000 Marseille, France; 11 Hospices civils de Lyon, Institute of Hematology and Oncology Pediatrics, F-69000 Lyon, France; 12 AP-HP, Department of Pediatric Hematology and Immunology, Robert Debre University Hospital, F-75019 Paris, France

**Keywords:** Cancer genomics, Paediatrics, Cancer epidemiology

## To the Editor:

Acute myeloid leukemia (AML) accounts for about 20% of acute leukemias in children and still has a poor prognosis compared to lymphoblastic leukemia (5-year survival rate about 68%) [1]. Over the years, knowledge of oncogenetic abnormalities has improved, allowing AML to be classified into groups with different prognosis, leading to different treatment intensity [2]. However, most genetic alterations have been described in adult cohorts. Bolouri et al. [3] and Marceau-Renaut et al. [4] have shown that the molecular landscape in children differs from that of adult AMLs and further studies are needed for a comprehensive classification of pediatric AML.

RUNX1, runt related transcription factor 1, is a transcription factor expressed in hematopoietic cells, plays a role in the early differentiation of progenitor and stem cells, and is known to be involved in hematologic diseases and leukemogenesis as a site of mutations [5].

In adult AML cohorts, *RUNX1* mutations are identified in 5–8% of younger patients, associated with M0 FAB subtype, normal karyotype and correlate with poor clinical outcome [6–8]. As an unfavorable marker, adult patients with *RUNX1* mutation are stratified in high risk group of treatment [9]. In contrast to *RUNX1* mutations, *RUNX1* deletions have rarely been studied and their impact is therefore unknown. In children, the impact of *RUNX1* mutations or deletions remains unclear because of their low frequency and is not used for risk stratification and choice of treatment intensity.

438 children with de novo AML were treated in the ELAM02 trial and *RUNX1* gene status was screened in 386 of them. *RUNX1* abnormality was found in 8% (29 of 386) of the cases, 24 patients with mutation and 5 with deletion. Because the majority of *RUNX1* mutations in AML behave as loss-of-function mutations, we decided to study both *RUNX1* mutations and deletions as one group.

Main clinical, cytological and cytogenetic characteristics of children with *RUNX1* mutated and deleted *(RUNX1*^*m/del*^*)* compared with *RUNX1* wild type (*RUNX1*^wt^) are reported in Table 1.

**Table 1:** Clinical, cytological, cytogenetic characteristics and outcome of RUNX1^m/del^ and RUNX1^wt^ children.

	*RUNX1*^m/del^	*RUNX1*^wt^	*P value*
	*n* = 29 (8%)	*n* = 357 (92%)	
*Clinical data, n (%)*			
Male gender	14 (48%)	196 (55%)	0.56
Age at diagnosis			
≤2 years old	2 (7%)	79 (22%)	0.06
Median age [min-max]	11.2 [0.7–17.2]	8.4 [0–18.6]	0.07
CNS involvement	3 (10%)	57 (16%)	0.60
White blood cells count at diagnosis			
>100 G/L	4 (14%)	57 (16%)	0.62
Median count	10.8 [1.7 –445]	17.7 (0.4–575)	0.20
*Risk group*			
Standard	2 (7%)	90 (25%)	
Intermediate	20 (69%)	181 (51%)	ns
Adverse	6 (21%)	84 (24%)	
*Cytogenetics, n (%)*			
Normal	10 (34%)	90 (25%)	0.28
CBF	2 (7%)	90 (25%)	0.02
MLL	0	78 (22%)	0.002
−7/del7q	5 (17%)	28 (8%)	0.09
Complex karyotype	6 (21%)	39 (11%)	0.13
Missing	1 (4%)	2 (1%)	0.21
*FAB classification, n (%)*			
M0	5 (17%)	19 (5%)	0.03
M1	8 (28%)	54 (15%)	0.11
M2	8 (28%)	82 (23%)	0.65
M4	3 (10%)	76 (21%)	0.23
M5	1 (3%)	85 (24%)	0.009
M6	2 (7%)	9 (3%)	0.20
M7	1 (3%)	18 (5%)	1
Unclassified + basophil	1 (3%)	9 (3%)	0.55
Chloroma	0	5 (1%)	1
*Outcome, n (%)*			
Complete remission	24 (83%)	327 (92%)	0.17
Relapse	11 (38%)	122 (34%)	0.69
HSCT	9 (31%)	99 (28%)	0.67
Death	17 (59%)	86 (24%)	<0.001
*Survival, [CI 95%]*			
5-years OS	33.6% [18.6–60.8]	75.7% [71.3–80.4]	<0.001
5-years EFS	32.5% [16.8–62.8]	61.4% [56.2–67.2]	0.003

Data presented as number (%), unless otherwise indicated.

*CNS* central nervous system, *FAB* French-American-British classification.

*CBF* Core binding factor define by inv(16) or t(8;21).

*HSCT* hematopoietic stem cell transplantation.

*OS* Overall survival, *EFS* Event-free survival.

There were no differences between *RUNX1*^*m/del*^ and *RUNX1*^wt^ regarding sex, age, white blood cell count, or central nervous system involvement.

*RUNX1*^*m/del*^ AMLs were more likely to be AML-FAB M0 (5/29 (17%) vs 19/357 (5%), *p* value = 0.03), and exclusive with AML-FAB M5. *RUNX1* mutations were associated with a normal karyotype (10/24 (42%) vs 90/357 (25%), *p* = 0.09) as previously described in adult studies [6, 8], exclusive with *KMT2A* (11q23) rearrangement, and rarely associated with Core Binding Factor (CBF) abnormalities as t(8;21)(q22;q22). Therefore, 69% of *RUNX1*^*m/del*^ patients were classified in the intermediate risk group. The distribution between risk groups was similar between *RUNX1*^*m/del*^ and *RUNX1*^wt^ patients leading to a comparable treatment intensity.

We identified 30 *RUNX1* mutations in 24 patients (6 of them cumulating 2 mutations), 16 of them in the RUNT Homology domain, and 8 mutations had been previously described in the literature by Brown et al. [10]. We found no association between the level of variant allele frequency (VAF), type (frameshift, missense, nonsense), and location of the mutation with leukemia prognosis. In addition to mutated patients, 5 patients had *RUNX1* deletion (between 50 kb and 1,6 Mb; all involving the RUNT domain).

Based on the 2015 American College of Medical Genetics and Genomics and Association for Molecular Pathology guidelines [11], all *RUNX1* alterations are classified as pathogenic or likely pathogenic except for 2; the first is known to be a benign variant of *RUNX1* gene and the second is of unknown significance. These 2 patients are cured and were treated in the favorable group because of CBF alteration.

*RUNX1* mutated patients had a higher number of co-mutations compared with the rest of the cohort (2.71 on average vs 1.43, *p* < 0.001), as described by Brown et al. [10]. The most common class of co-mutated genes involved control kinase signaling (50%) especially *FLT3-ITD*, *NRAS, FLT3-TKD and KRAS or WT1*. *RUNX1* alterations were also associated with *EZH2* and *BCOR* mutations, as reported by Gaidzik [8] in adults.

We found no *CEBPA*, *NPM1, TET2, SETBP1, RAD21, CBL* mutations in *RUNX1* mutated patients. Except for 1 patient with *RUNX1* deletion who had a co-mutation in *U2AF1, RUNX1* mutated patients had no alteration in splicing factor (SF) (such as *SRSF2* or *SF3B1*). In contrast to what was recently reviewed by Inge van der Werf et al. [12] in adult AML, the prognostic value of *RUNX1* mutations in our cohort was not limited to their co-occurrence with SF mutations.

**Fig. 1:**
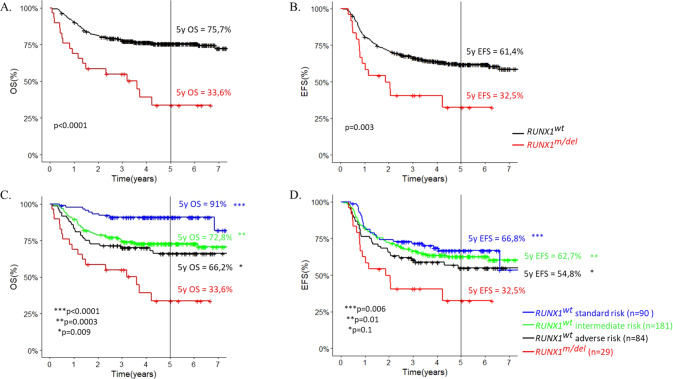
Childhood AML outcome according RUNX1 status. **A** Overall survival according RUNX1 status - **B** Event-free survival according RUNX1 status - **C** Overall survival according to risk group and RUNX1^m/del^ - **D** EFS according to risk group and RUNX1^m/del^ . *Significant difference between RUNX1^m/del^ and RUNX1^wt^ risk subgroup.

We observed a significantly worse outcome for *RUNX1*^*m/del*^ patients compared with *RUNX1*^wt^ (5-year EFS = 32.5% [95% confidence interval = 16.8–62.8] vs 61.4% [CI = 56.2–67.2]; and 5-yOS = 33.6% [CI = 18.6–60.8] vs 75.7% [CI = 71.3–80.4]). Hazard ratios for EFS and OS were 2.2 (CI = 1–4,7; *p* value = 0.003) and 3.3 (CI = 1.4–7.5; *p* < 0.0001), respectively. Comparing by risk groups, *RUNX1*^*m/del*^ patients still had a worse outcome than patients in adverse risk group (5-y OS = 33.6% for *RUNX1*^*m/del*^ vs 66.2% for *RUNX1*^wt^ in adverse risk group). (Fig. 1).

However, *RUNX1*^*m/del*^ patients achieved complete remission (CR) as *RUNX1*^wt^ patients (83% vs 92%, *p* = 0.17) and had the same relapse rate (11/29 (38%) vs 122/357 (34%), *p* = 0.69).

Causes of death included leukemia (7/17), infection (4/17), or post-transplant toxicity (4/17). To elucidate the reason for this high toxicity and to understand how the *RUNX1* alteration leads to such a poor outcome, further studies need to be performed on a larger cohort.

Three arguments led us to question whether some of these children with mutated *RUNX1* might have a constitutional mutation: (1) the high toxic death rate, which could be explained by abnormal hematopoiesis exacerbating treatment toxicities(2), the high number of patients (16/24, 67%) with an increased VAF (>30%), suggesting a possible germline origin, and (3) the presence of two different *RUNX1* mutations in 6 patients, one of which may be of germline origin and a second of somatic origin [13].

In our study, among these 16 patients with VAF > 30%, only 1/12 of the patients tested was confirmed to have a *RUNX1* germline mutation; this patient had only one *RUNX1* mutation, is still alive, and showed no treatment-related toxicity.

Among the 5 deletions, only one patient had a large germline *RUNX1* deletion (1.6 Mb), already described by Preudhomme et al. [14].

Although further studies are needed to determine whether these constitutional mutations require specific treatment, the systematic search for germline mutations in complete remission may be of interest to adjust therapeutic agents.

In conclusion, our study demonstrates the prevalence, co-mutation profile, and poor survival of *RUNX1-*mutated or -deleted AML in a well-described pediatric cohort. The *EZH2* and *BCOR* genes, known as chromatin modifiers, are frequent co-mutations in *RUNX1*^*m/del*^ leukemia and may play a role in the unfavorable future of this leukemia. Considering other pediatric studies [3, 15], *RUNX-* mutated and -deleted AML in children should be classified into a poor risk group to benefit from optimal intensified treatment, taking into account the high mortality due to toxicity.

## Supplementary information

Supplemental data

## Data Availability

The datasets analyzed during the current study are available from the corresponding author on reasonable request.
