# Supplementary material for: Prognostic impact of RUNX1 mutations and deletions in pediatric acute myeloid leukemia: results from the French ELAM02 study group
Source: Leukemia. 2023 Jun 16;37(8):1723–6. doi: 10.1038/s41375-023-01931-y (PMC10400410; doi:10.1038/s41375-023-01931-y)
Supplement: Supplementary file 1 — Supplemental data [file 41375_2023_1931_MOESM1_ESM.docx]

**Supplemental data**

**Supplemental Table S1 –** Details of Clinical and Molecular characteristics of patients with RUNX1 mutation or deletion

**Supplemental Table S2 –** Molecular landscape by NGS in RUNX1^m/del^ and RUNX1^wt^ patients

**Supplemental Figure S1 –** Representation of RUNX1 mutation and deletion

**Supplemental Figure S2 –** RUNX1 co-mutation by cytogenetic sub-group

**Supplemental Text –** Patients and methods

**Supplemental Table S1 –** Details of Clinical and Molecular characteristics of patients with RUNX1 mutation or deletion

| Id patient | Status  after  conso 1 | HSCT after CR1 | Relapse | Status | Cause of death | Risk group  (1: favorable  2:intermediate  3: adverse) | *FLT3-ITD* | *NUP 98* fusion | *RUNX*1-VAF (%) | *RUNX1* abnormality after CR | % blast in BM aspiration J0 | | Mutation / Deletion | ACMG-AMP clinical significance  (1,2: no impact  3: unknown,  4,5: pathogenic) |
| --- | --- | --- | --- | --- | --- | --- | --- | --- | --- | --- | --- | --- | --- | --- |
| 1 | CR | Yes | No | Alive | - | 2 | 0 | 0 | 20 | - | | 97 | c.653_656delGCTTinsCCTGGG : p.Ser218Thrfs*20 | LP (4) |
| 2 | CR | Yes | No | Alive | - | 2 | 0 | 0 | 38 | No | |  | c.1170_1180delAGCGCAGGGAG:p.Ala391Profs*205 | P (5) |
| 3 | CR | No | Yes | Alive | - | 3 | 0 | 0 | 28 | - | | 84 | c.314A>G:p.His105Arg | P (5) |
| 4 | CR | Yes | No | Alive | - | 2 | 0 | 0 | 38//43 | No | | 90 | c.665_666dupCC:p.Glu223Profs*15//c.1352_1353insGATAATTAGTA:p.Asp451Glufs*147 | P (5) // LP (4) |
| 5 | CR | Yes | No | Alive | - | 2 | 0 | 0 | 4 | - | | 35 | c.424_425insGCG:p.Ser141_Ala142insGly | P (5) |
| 6 | CR | No | No | Alive | - | 1 | 0 | 0 | 38 | No | | 39 | c.1098_1103dupCGGCAT:p.Ile366_Gly367dup | B (1) |
| 7 | CR | No | No | Alive | - | 2 | 0 | 0 | 34 | No | | 40 | c.283C>T:p.Pro95Ser | P (5) |
| 8 | CR | No | No | Alive | - | 2 | 0 | 0 | 56 | NE | | 20 | c.1184C>G:p.Pro395Arg | VUS (3) |
| 9 | CR | No | Yes | Alive | - | 1 | 1 | 0 | 44//46 | No | | 80 | c.453G>T:p.Met151Ile//c.697C>T:p.Arg233Cys | LP (4) // VUS (3) |
| 10 | No | No | No | Alive | - | 2 | 0 | 0 | 55 | Yes | | 18 | c.601C>T:p.Arg201* | P (5) |
| 11 | CR | Yes | No | Alive | - | 3 | 0 | 0 | 14//13 | - | | 64 | c.958C>T:p.Arg320*//c.1036dupC:p.Arg346Profs*254 | P (5) // P (5) |
| 12 | CR | No | Yes | Deceased | Leukemia | 2 | 1 | 1 | 25 | - | | 95 | c.749_750insAG:p.Ala251Valfs*4 | LP (4) |
| 13 | CR | No | Yes | Deceased | Post allograft toxicity | 2 | 1 | 0 | 42 | No | | 65 | c.602G>A:p.Arg201Gln | P (5) |
| 14 | CR | No | Yes | Deceased | Leukemia | 3 | 0 | 0 | 36 | NE | | 84 | c.427_428insGGCTCGGCTG:p.Glu143Glyfs*20 | P (5) |
| 15 | CR | No | Yes | Deceased | Post allograft toxicity | 2 | 1 | 0 | 33 | No | | 95 | c.492_493insCCTAACCA:p.Gly165Profs*14 | P (5) |
| 16 | No | No | No | Deceased | Leukemia | 2 | 0 | 0 | 43 | NE | | 38 | c.602G>A:p.Arg201Gln | P (5) |
| 17 | Death | No | No | Deceased | Infection (candidemia) | 3 | 0 | 0 | 16//14 | - | | . | c.421T>C:p.Ser141Pro//c.610C>T:p.Arg204* | P (5) // P (5) |
| 18 | CR | No | Yes | Deceased | Infection | 2 | 1 | 0 | 37 | No | | 95 | c.908C>G:p.Ser303* | P (5) |
| 19 | CR | Yes | Yes | Deceased | Leukemia | 2 | 0 | 0 | 38 | No | | 91 | c.456_458dupGAA:p.Lys152dup | P (5) |
| 20 | Death | No | No | Deceased | Infection (sepsis) | 2 | 0 | 0 | 20 | - | | 10 | c.1090_1103del:p.Ile364Valfs*231 | P (5) |
| 21 | CR | Yes | No | Deceased | Post allograft toxicity | 2 | 1 | 1 | 40 | No | | 92 | c.279_280dupCA:p.Ser94Thrfs*29 | P (5) |
| 22 | CR | No | No | Deceased | Infection (mucormycosis) | 2 | 0 | 0 | 42//45 | NE | | 87 | c.610C>T:p.Arg204*//c.619C>T:pArg207Trp | P (5) // LP (4) |
| 23 | CR | No | No | Deceased | Complications of Fanconi | 2 | 0 | 0 | 11 | - | | 45 | c.1256_1262dupTGGGCGG:p.Glu422Glyfs*180 | P (5) |
| 24 | CR | No | No | Deceased | Infection (Aspergillus) | 3 | 0 | 0 | 33//33 | No | | 21 | c.422C>T:p.Ser141*//c.493G>A:p.Gly165Ser | P (5) // P (5) |
| 25 | CR | Yes | No | Alive | - | 3 | 0 | 0 | - | No | | 94 | Exon 3-4-5-6-7 deletion (115kb) | P (5) |
| 26 | Death | No | No | Deceased | Toxicity | 2 | 0 | 0 | - | No | | 66 | Exon 3-4 deletion (50kb) | P (5) |
| 27 | CR | No | Yes | Deceased | Leukemia | 2 | 0 | 0 | - | No | | 90 | Exon 3 deletion (160kb) | P (5) |
| 28 | CR | No | Yes | Deceased | Leukemia | 2 | 0 | 0 | - | No | | 73 | Exon 3-4-5-6-7-8-9 deletion (332kb) | P (5) |
| 29 | CR | Yes | Yes | Deceased | Leukemia | 2 | 0 | 0 | - | Yes | | 21 | *RUNX1* deletion (1.6Mb) | P (5) |
|  |  |  |  |  |  |  |  |  |  |  | |  |  |  |

*HSCT : hematopoietic stem cell transplantation, CR1 : complete remission after consolidation, BM : bone marrow*

*VAF : variant allele frequency of RUNX1 mutation in bone marrow aspiration at diagnosis. When two different mutations were founded, each frequence is written XX//XX.*

*When VAF < 30%, mutation was considered somatic.*

*0 : no mutation, 1 : presence of mutation, NE : not evaluated*

*B: benign, VUS: variant of uncertain significance, LP : likely pathogenic, P : pathogenic*

**Supplemental Table S2-** Molecular landscape by NGS in RUNX1^m/del^ and RUNX1^wt^ patients

|  |  | RUNX1^m/del^ | | RUNX1^wt^ | |  |
| --- | --- | --- | --- | --- | --- | --- |
|  |  | **29** | 8% | **357** | 92% | **p-value** |
| Molecular profile | FLT3-ITD | 6 | (21%) | 51 | (14%) | 0.41 |
|  | NRAS | 5 | (17%) | 97 | (27%) | ns |
|  | FLT3-TKD | 4 | (14%) | 30 | (8%) | ns |
|  | WT1 | 3 | (10%) | 38 | (11%) | ns |
|  | BCOR | 3 | (10%) | 2 | (1%) | 0.004 |
|  | EZH2 | 3 | (10%) | 7 | (2%) | 0.03 |
|  | NUP98 | 2 | (7%) | 8 | (2%) | ns |
|  | KIT | 2 | (7%) | 46 | (13%) | ns |
|  | KRAS | 2 | (7%) | 45 | (13%) | ns |
|  | ETV6 | 2 | (7%) | 5 | (1%) | ns |
|  | BCORL1 | 2 | (7%) | 4 | (1%) | ns |
|  | PHF6 | 2 | (7%) | 12 | (3%) | ns |
|  | IDH1 | 1 | (3%) | 11 | (3%) | ns |
|  | IDH2 | 1 | (3%) | 5 | (1%) | ns |
|  | DNMT3A | 1 | (3%) | 3 | (1%) | ns |
|  | GATA2 | 1 | (3%) | 15 | (4%) | ns |
|  | ASLX1 | 1 | (3%) | 8 | (2%) | ns |
|  | SMC1A | 1 | (3%) | 4 | (1%) | ns |
|  | PTPN11 | 1 | (3%) | 23 | (6%) | ns |
|  | TP53 | 1 | (3%) | 3 | (1%) | ns |
|  | SMC3 | 1 | (1%) | 5 | (1%) | ns |
|  | JAK2 | 1 | (3%) | 10 | (3%) | ns |
|  | U2AF1 | 1 | (3%) | 5 | (1%) | ns |
|  | NPM1 | 0 | (0%) | 36 | (10%) | ns |
|  | CEBPA | 0 | (0%) | 16 | (4%) | ns |
|  | SRSF2 | 0 | (0%) | 2 | (1%) | ns |
|  | SF3B1 | 0 | (0%) | 1 | (0%) | ns |
|  | number of mutations | 2,71 | | 1,43 | | <0,001 |

**Supplemental Figure S1 –** Representation of RUNX1 mutation and deletion


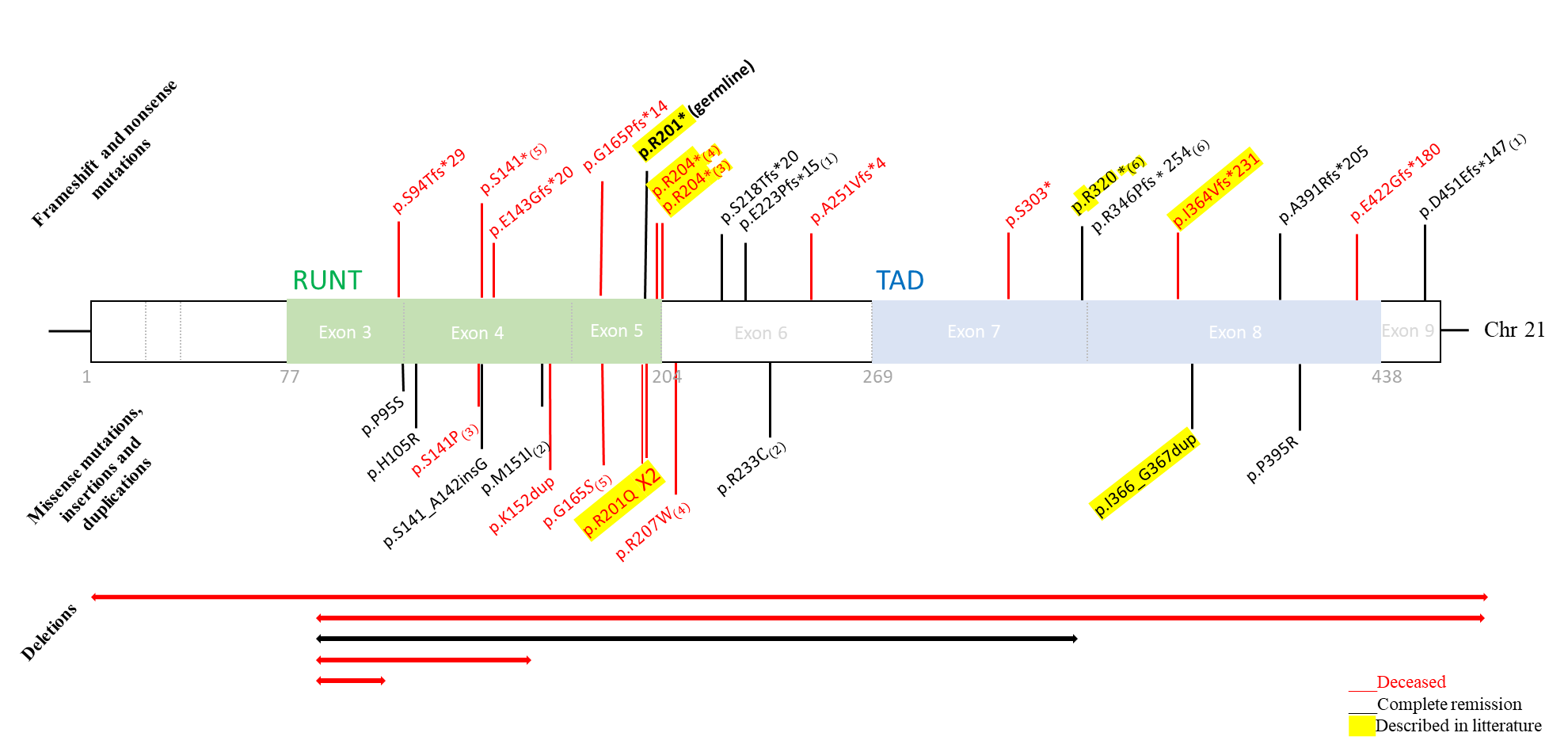


Fs : frameshift

* nonsense mutation

(x) two mutations presented by a single patient

TAD : Transactivation domain

Mutations described by Brown et al.^10^

**Supplemental Figure S2 –** RUNX1 co-mutation by cytogenetic sub-group

|  | t(8;21)(qq22;q22) |  | Normal Karyotype |  | Adverse Karyotype |  | other intermediate Karyotype |
| --- | --- | --- | --- | --- | --- | --- | --- |

***one column per patient***

**Supplemental text - Patients and methods**

**Patients**

We studied children treated in the ELAM02 trial (Treating Patients With Childhood Acute Myeloid Leukemia With Interleukin-2; ClinicalTrials.gov NCT00149162), aged between 0 and 18 years old with newly diagnosed AML. They were enrolled between March 2005 and December 2011 in 28 centers in France. Acute promyelocytic leukemia, therapy-related AML and Down syndromes were excluded from the ELAM02 trial. Patient selection was based on the availability of genomic DNA at AML diagnosis. They were classified into three risk groups depending on cytogenetics and molecular fusion transcripts at diagnosis: t(8;21)(q22;q22), inv(16)(p13;q22) or t(16;16) were favorable. Monosomy 7, 5q abnormalities, t(6;9)(p23;q34), t(6;11)(q27;q23), inv(3)(q21;q26) or t(3;3)(q21;q26) and complex karyotype defined by the presence of 3 or more unrelated chromosome abnormalities were classified in adverse group. The others were intermediate. Treatment consisted of an induction course associating mitoxantrone and continuous intravenous standard dose cytarabine followed by a first course of consolidation including high dose cytarabine combined with amsacrine. Complete remission was defined by less than 5% blasts in bone marrow aspiration with normal maturation of all cellular components and blood cell count within normal ranges after one consolidation. Afterwards, depending on the risk group of their disease and the existence of a matched related hematologic stem cell (HSC) donor, children either received 2 more consolidation courses or allogenic HSC transplant.

The study was approved by the Ethics Committee of Saint-Antoine Paris University Hospital (Assistance Publique- Hôpitaux de Paris) and by the Institutional Review Board of the French Regulatory Agency and was conducted in accordance with the Declaration of Helsinki.

**Genomic analysis**

Exhaustive mutation profile was retrospectively performed on genomic DNA from bone marrow aspiration at diagnostic and complete remission when needed studied by high-throughput sequencing (HTS). The panel studied 36 genes recurrently mutated in myeloid malignancies, including genes encoding proteins involved in kinase signaling [*CBL* (exons 8-9), *FLT3* (exon 20), *JAK2* (exons 12, 14, 16), *KIT* (exons 8-13, 17), *KRAS* (exons 2-3), *MPL* (exon 10), *NRAS* (exons 2-3), *PTPN11* (exons 3, 13), *SETBP1* (exon 4)], transcription factors [*CEBPA* (exon 1), *ETV6* (exons 1-8), *GATA1* (exon 2), *GATA2* (exons 2-6), *RUNX1* (exons 1-6)], tumor suppressors [*PHF6* (exons 2-10), *PTEN* (exons 5-7), *TP53* (exons 2-11), *WT1* (exons 7, 9)], chromatin modifiers [*ASXL1* (exons 11-12), *BCOR* (exons 2-15), *BCORL1* (exons 1-12), *EZH2* (exons 2-20)], DNA methylation [*DNMT3A* (exons 2-23), *IDH1* (exon 4), *IDH2* (exon 4), *TET2* (exons 3-11)], cohesin complex [*NIPBL* (exons 2-47), *RAD21* (exons 2-14), *SMC1A* (exons 1-25), *SMC3* (exons 1-29), *STAG2* (exons 3-35)], RNA splicing [*SF3B1* (exons 13-18), *SRSF2* (exon 1), *U2AF1* (exons 2, 6), *ZRSR2* (exons 1-11)] and *NPM1* (exon 11), as previously described (Marceau-Renaut et al).^4^

A high depth of coverage (>1500x) was obtained for all genes, allowing detection of mutations with a variant allele frequency (VAF) as low as 1%. The list of genes is described in Annex 1. For patients with VAF >30% and available DNA samples in complete remission, *RUNX1* mutation was screened by direct Sanger sequencing. The search for the *FLT3*-internal tandem duplication (ITD) was performed for all patients by fragment analysis as previously described.

Genomic DNA from bone marrow aspiration was also hybridized to Cytoscan HD array (Affymetrix) according to the manufacturer’s protocol at diagnosis and complete remission when needed. Data were analyzed using the Chromosome Analysis Suite (ChAS) software (Affymetrix). Deletions of *RUNX1* were annotated for size, position and location based on the human genome version 19 (hg19) of the UCSC Genome Browser.

**Statistical analysis**

Comparisons between patient subgroups were performed by the Mann-Whitney test for continuous variables and by Fisher’s exact test for categorical variables. All P-values were two-sided and values <0.05 were considered statistically significant. Event-free survival (EFS) and overall survival (OS) were estimated by the Kaplan-Meier method and compared by Log-rank test. Hazard ratios (HRs) are given with 95% confidence interval (CI). EFS was measured from the date of complete remission to the date of first event (relapse, death) or to the date of last follow-up. OS was measured from the date of diagnosis to the date of death from any cause or last follow-up. All statistical tests were performed with GraphPad Prism 9 and R3.2.3 software packages (R Development Core Team, Vienna, Austria).
